# From Risk Assessment to Intervention: A Systematic Review of Thrombosis in Plastic Surgery

**DOI:** 10.7759/cureus.41557

**Published:** 2023-07-08

**Authors:** Heli S Patel, Justin M Camacho, Anastassia Shifchik, Jacob Kalmanovich, Emma Burke, Salam Harb, Alan Patrus, Daniel Cheng, Amir Behnam

**Affiliations:** 1 Allopathic Medicine, Nova Southeastern University Dr. Kiran C. Patel College of Allopathic Medicine, Davie, USA; 2 Department of Medicine, Drexel University College of Medicine, Philadelphia, USA; 3 Medicine, Drexel University College of Medicine, Philadelphia, USA; 4 Allopathic Medicine, Nova Southeastern University Dr. Kiran C Patel College of Allopathic Medicine, Davie, USA; 5 Plastic and Reconstructive Surgery, Tower Health Medical Group, Wyomissing, USA; 6 Plastic and Reconstructive Surgery, Tower Health Medical Group, Wyosmissing, USA

**Keywords:** caprini risk assessment, intervention, plastic surgergy, thromboembolism, thrombosis

## Abstract

Thromboembolism is a feared complication in plastic surgery and is linked to higher rates of morbidity and mortality. Despite extensive research, there is a lack of consistency between recommendations and clinical protocols to be implemented pre and post-surgery to reduce the incidence of thromboembolism. A systematic literature review was conducted using Pubmed and Scopus databases to determine the risk factors, screening methods, and existing treatment models for thromboembolism prevention. Articles in non-English languages were excluded. Analysis indicated that predominant risk factors include age (>35), elevated body mass index, coagulation disorders, smoking, estrogen therapies, genetic predisposition, vascular endothelium damage, stasis, and use of general anesthesia in patients with a history of cancer. Implementation of a proper prophylactic protocol is dependent on understanding the interplay between the aforementioned risk factors and the utilization of well-defined, evidence-based guidelines, such as the 2005 Caprini Risk Assessment Model and ultrasound surveillance. The literature review revealed that mechanical prophylaxis is the primary prevention method, followed by thromboprophylaxis for patients with higher Caprini scores. Plastic surgeons often underestimate the present risk stratification tools available for the prophylactic intervention of thromboembolism due to the fear of bleeding or hematoma complications postoperatively. In summary, this literature review emphasizes the importance of plastic surgeons selecting protocols that is inclusive of the patient's risk profile to yield a reduced risk of thromboembolism.

## Introduction and background

Thrombosis is the development of a blood clot that can obstruct either the arterial or venous blood vessels. Notably, Virchow's triad has been used to describe several mechanisms that can lead to a thrombotic event, which include: endothelial damage, hypercoagulability, and blood stasis [1]. Thrombosis is a dreaded and feared complication that many surgeons use thromboprophylaxis measures based on a patient's overall health and pre-existing conditions. While many cases are asymptomatic, it can ultimately result in the death of a patient when the thrombus dislodges from its primary location and travels systemically to cause blockage at a secondary location. Increased systemic risks, such as thrombosis leading to pulmonary embolism complications, are further coupled with procedural-specific risks, including microvascular anastomosis thromboses in free-flap procedures [2]. Moreover, thrombosis increases the risk of cerebrovascular accidents and myocardial infarction by two folds [1].

The key to preventing thromboembolic events is recognizing risk factors that promote thrombus development. Current literature elucidates that the risk of thrombosis is elevated in individuals who have several risk factors, which include but are not limited to, increased age, elevated body mass index, hypercoagulability, family history, smoking, and estrogen therapy [3]. Plastics and reconstructive surgery, oncological surgery, pelvic surgery, and orthopedic surgery present the highest thrombogenic incidence amongst surgical specialties [4]. 

Two prominent risk factors highly applicable to surgical procedures include anesthesia, which promotes a prothrombotic state, and trauma due to surgery, which results in an inflammatory response inducing secondary thrombocytosis due to elevated levels of prothrombin and fibrinogen [5]. For instance, Kubo et al. mention that 5-30% of all free-tissue transfers and implantations are complicated by thrombosis [2]. While flaps with higher vascular resistance thrombose more frequently, Forte et al. emphasized decreased complications in flaps with higher blood flow velocities [6]. This statistic is amongst many alarming findings involving thrombosis within plastic surgery procedures. 

It becomes essential to understand the physiological principles and the mechanism of thrombosis as a means to avoid patient complications and develop better protocols for prevention. Therefore, this review aims to highlight the current literature on thrombotic complications within plastic surgery procedures by providing a comprehensive overview of its epidemiology, diagnostic techniques, preventative measures, and treatment options, along with emphasizing areas that require further research for quality improvement.

## Review

Methods 

**Figure 1:**
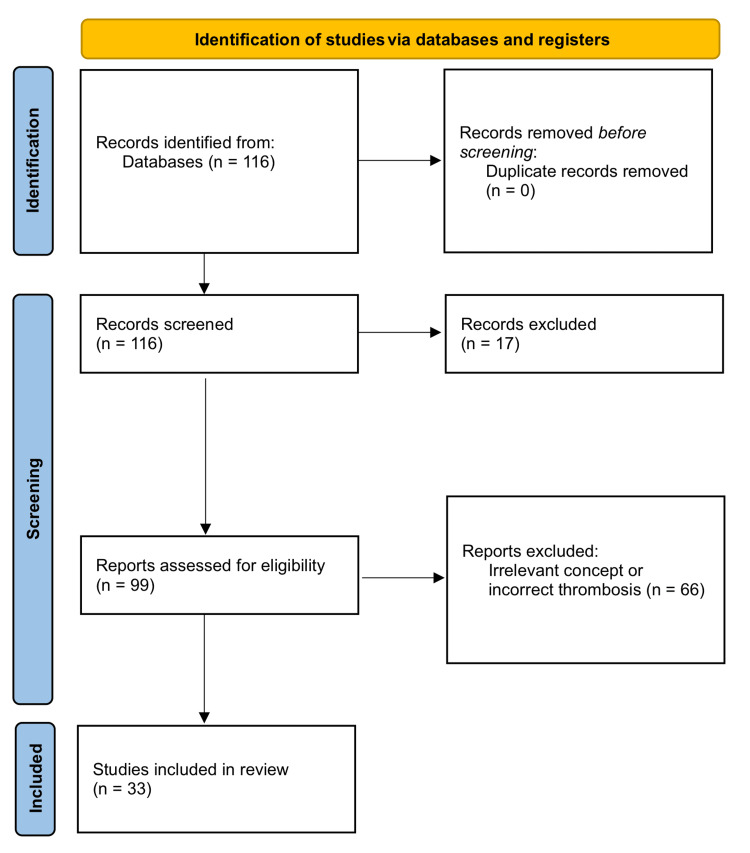
PRISMA flow diagram of studies included in the review

This systematic review was performed using the PubMed database for articles pertaining to thrombosis as a complication during plastic and reconstructive surgery. A comprehensive search through the database was conducted using the terms "thrombosis", "complications", "plastic surgery" and "reconstructive surgery." After screening the titles and abstracts, the articles were identified for free-full texts. Additional articles were screened after reviewing references from previously identified articles. The search strategy was designed to include all types of literature, including clinical trials, cohort studies, retrospective studies, case studies, and systematic reviews. Articles published in English in any journal were considered. Preferred Reporting Items for Systematic Reviews and Meta-Analyses (PRISMA) guidelines were followed (Figure 1). Data related to epidemiology, surgical procedures, assessment criteria, prophylactic plans, and treatments were extracted. After a review of the free-full texts, a total of 116 articles were reviewed in detail. Of these articles, 33 met the inclusion criteria. The remaining articles were excluded since they did not comment on thrombosis as a complication in relation to plastic or reconstructive surgery.

Results 

Risk Factors of Thrombosis

Each component of Virchow's triad contributes to the formation of a thrombus [7-8]. In the field of plastic surgery, long procedures such as free flap procedures tend to have a higher incidence of reperfusion injuries, which lead to intravascular wall damage due to the formation of reactive oxygen species. As a result of the endothelial damage, compromised areas become subsequently exposed to hypoxic conditions, which set the stage for microvascular thrombosis by inducing pro-coagulation and disruption of vascular integrity due to microsurgical errors [9].

Clinically, stasis may also manifest due to immobilization during surgery and the sedentary requirements for the patient post-operatively. The length of surgeries is one of the foremost factors influencing surgical immobilization with free-flap procedures lasting up to three to six hours [10]. Intraoperative positioning can result in a subsequent reduction of blood flow, which heightens the risk of a patient experiencing thrombosis during the procedure [11]. For example, many vaginal surgeries require the patient to utilize a lithotomy position which has been shown to increase the risk of thrombosis due to decreased venous drainage [12]. Additionally, immobilization during recovery further increases risk by promoting hemostasis and decreasing fibrinolytic activity [11]. 

Hypercoagulable states generally involve intrinsic factors that alter blood composition or vascular functionality. This includes hematological disorders such as polycythemia vera, thrombophilia, hemodynamic disorders, or even [7]. Beiben et al. exemplify that hypercoagulable patients have a 13% thrombosis incidence compared to 6.3% in non-hypercoagulable patients [13]. Hypercoagulable states also occur with genetic causes such as Factor V Leiden mutation. This mutation disrupts the natural anticoagulation properties of protein C, which has been shown to cause 20-40% of venous thrombosis [8]. Other genetic causes include antithrombin III deficiency, protein C, and protein S deficiency [8]. 

In addition to risk factors stemming from Virchow's triad, age, obesity, and body mass index (BMI), as well as other minor factors, have been shown to increase thrombosis. Age is a contributing factor; however, physiologic age rather than absolute age greatly affects thrombosis risk. The physiologic age is altered with comorbidities [14]. Obesity is a high-risk factor alongside atherosclerosis, hypertension, hyperlipidemia, and diabetes [14-15]. ElAbd et al. elucidate that in a cohort of obese patients (BMI >30 kg/m2) and diabetic patients, the total complication rate increased to 53.4% and 62.5%, respectively [15]. 

Minor risk factors include the male sex (33.3% major complication risk compared to 14.9% in females) [15], antiphospholipid antibody syndrome [14], hormonal therapy [16], and use of tamoxifen, specifically in the first two years of exposure to the therapy [11]. While ElAbd et al. exemplify the increased risk of minor complications in smokers (39.5% vs. 23.8% nonsmokers), there is uncertainty regarding the correlation of smoking with increased thrombosis risk [15]. Smoking has the potential to cause endothelial damage and lead to pro-coagulable states; however, the current deep venous thrombosis (DVT) assessment tools do not include it as a factor [17].

Epidemiology of Thrombosis in Plastic Surgery

Thromboembolism (TE) continues to be a daunting complication that plagues many surgical specialties, including plastic surgery. One study has shown that there is an increased incidence of thromboembolism in several procedures, including breast surgery (36%), combined surgery (24.3%), head and neck (18%), abdominoplasty (11.5%), and liposuction (10%) [4]. 

Within the post-bariatric patient cohort (patients who have lost greater than 50 lbs), those undergoing body contouring procedures such as abdominoplasties and liposuction have been shown to have a higher risk profile for venous thromboembolism from 0.2% to 9.4% [18-19]. Abdominoplasties, in particular, have the highest incidences of venous thromboembolism (VTE) due to the increased disruption of superficial veins in conjunction with the amount of surface area involved in the procedure [18]. These factors, coupled with extensive operation time and post-operative stasis, establish the potential for thrombus formation [18-19]. Gravante et al. found that operations longer than 140 minutes had a relative risk of 3.0 with an 8.8% increase rate of pulmonary embolism (PE) [20]. Additionally, the location may also play a role in thrombosis risk. Truncal procedures have a statistically greater chance of deep vein thrombosis (DVT)/PE complications compared to extremity body contouring [21].

The literature surrounding thrombosis risk in the breast reconstruction patient remains non-homogeneous and dependent on the type of reconstruction performed [21-22]. In addition to common surgical complications such as infection, seroma, flap loss, fat necrosis, and hematoma, Hershenhouse et al. found that 11/44 studies analyzed demonstrated vessel thrombosis in reconstructed breast flaps [22]. While statistically insignificant, a comparison of immediate and delayed reconstruction revealed that flap loss and flap vessel thrombosis occurred at lower levels following immediate reconstruction [22, 23]. 

Free flap surgeries are also prone to increased incidences of specifically venous thrombosis [24-25]. In their meta-analysis, Riot et al. mention that venous thromboembolism (VTE) occurred in 61.7% of cases compared to 38.3% of arterial thrombosis [24]. However, performing a second venous anastomosis during the procedure decreased venous thrombosis risk, reducing the risk of flap failure, likely due to the increased surface area provided in the case of microthrombi formation. Riot et al. mention that 3.1% of 3299 patients developed thrombosis in the single-anastomosis group compared to 2.3% of 1326 patients in the double-anastomosis group [24]. 

Thrombosis Assessment 

The Caprini Risk Assessment Model (RAM) is most often used to stratify individuals' risk of VTE [12]. Other risk assessments include the Davison model and Padua model, which are involved in non-surgical cases [4, 16]. The Caprini RAM has the greatest validation and has two iterations: 2005 and 2010. Caprini 2010 RAM updated the categorical placement and point assignments for several risk factors based on a novel published literature; however, the 2005 model has been demonstrated to have better VTE predictive capacity in plastic surgery procedures [5, 26]. Table 1 (Pannucci, 2012) provides a comparison of both models and illustrates the modifications in the updated iteration [26].

**Table 1:** Differences between the 2005 and 2010 versions of the Caprini Risk Assessment Models

Risk Factor	2005 Caprini Model	2010 Caprini Model
Operative time	0-44 minutes=1 point	0-59 minutes=1 point
	≥45 minutes=2 points	60-119 minutes=2 points
	-	120-179 minutes=3 points
		≥180 minutes=5 points
Body mass index (kg/m2)	≥25=1 point	≥30 & <40=1 point
	-	≥40 & <50=2 points
		≥50=3 points
Superficial venous thrombophlebitis (SVT)	Not a risk factor	History of SVT=3 points
Cancer	History of cancer=2 points	History of cancer=2 points
	Current cancer=2 points	Current cancer=3 points

When applied to the same patient cohort, those stratified with the 2005 model revealed improved risk stratification. Specifically, those stratified as "super-high" risk patients (Caprini score > 8) were more likely to have a 60-day VTE in the 2005 model compared to the 2010 model [26]. This is potentially due to the systematic increase of risk scores in the 2010 model which dilutes the high-risk stratification [26]. Caprini RAM uses a numerical system to rate each factor between one to five points to provide a risk value for VTE, as shown in Table 2 [4, 5, 16, 27, 28]. 

**Table 2:** VTE Incidence by Caprini RAM scores

Caprini Risk Assessment Model Designations		
Designation	Point Value	VTE Incidence
Low-Risk	<3	-
Moderate to High Risk	3-4	58%
High Risk	5-6	29%
Higher Risk	>6	13%

The Caprini RAM has several limitations, the greatest of which is that its development was not based on rigorous statistical methods nor based on plastic surgery outcomes [17, 27]. It was developed as an odds ratio analysis from a retrospective analysis of surgical, medical, and trauma patients [17]. Statistical comparisons between patient groups are challenging to produce with this criteria due to the lack of normal score distribution and non-linear nature [28]. Nonetheless, validation studies have supported the continued use of Caprini RAM scores in plastic surgery.

Diagnosis

Diagnosing deep vein thrombosis (DVT) following plastic surgery is often precipitated by post-operative monitoring, which includes assessing body temperature, skin color, signs of edema, and fluidity of the vasculature using a Doppler ultrasound [29]. Extensive research has been conducted to determine the safest and most efficacious method of detecting thromboembolisms. In particular, the Doppler ultrasound has been deemed as the gold standard for diagnosing DVT in plastic surgery outpatients, as it poses a 97% sensitivity and 94% specificity for patients who are clinically symptomatic for a DVT [28].

Furthermore, doppler ultrasound serves as a beneficial pre-operative screening tool by reducing the time required to evaluate a patient's risk factors for thrombosis and to evaluate the need for thrombotic chemoprophylaxis. On the other hand, color Doppler imaging can provide more detailed information because of the ability to visualize calf veins with a greater sensitivity to detect more precise clot locations and characteristics [28]. 

Prevention

To prevent the occurrence of thrombosis in high-risk plastic surgery procedures, various methods are utilized, including prophylactic treatment, monitoring time under surgery/ anesthesia, utilization of compression garments, mobility, and post-procedural monitoring. In this review, the primary strategy to prevent thrombosis involved the implementation of prophylactic protocols. Somogyi and colleagues found that the most significant of their recommendations in reducing the risk of VTE in abdominoplasties was the administration of celecoxib (Celebrex) 200 mg one hour before surgery [5].

Despite the known incidence of DVT in surgical procedures, Poore et al. exemplify the significant disparity in the utilization of pharmacologic prophylaxis by plastic surgeons [30]. For plastic surgeons performing facelifts, liposuctions, and combined procedures, prophylactic thrombosis measures are utilized at 45.7%, 43.7%, and 60.8%, respectively, in all the procedures [30]. In order to establish a widespread reduction in venous thrombosis and pulmonary embolism in plastic surgery, it is recommended for surgeons to follow protocols published in Plastic and Reconstructive Surgery by Davison and colleagues and reviewed by Horton et al. to maintain patient safety [30-33]. 

The length of surgery and types of anesthesia are key areas that can be modified to prevent thrombosis in several types of plastic surgeries. Local anesthesia offers a safer alternative to general anesthesia by limiting minor and major complications [33]. Furnas et al. found that utilization of local anesthetic in labiaplasty procedures is ideal because it avoids complications of general anesthesia like nausea and vomiting, aspiration pneumonia, malignant hyperthermia, and thromboembolic events [12, 33]. In liposuction, anesthesia is an extremely important factor for the success and safety of the procedure [5]. Lidocaine is used most commonly as the anesthetic agent for subcutaneous infiltration since it has a wider margin of safety than Marcaine and is more easily reversed [34]. Historically, the recommended dose of lidocaine is less than 7 mg/kg; however, in liposuction procedures, there are several factors that allow for increased administration. This is because of decreased risk of systemic toxicity due to the slow absorption of anesthetic from fat, persistent vasoconstriction from epinephrine, and removal of lidocaine in the liposuction aspirate. 

Compression is another method of prevention widely adopted by plastic surgeons. A study found that compression garments significantly slowed blood flow in the femoral vein, especially when patients were placed in the Fowler position [35]. As well as positioning during surgery, the stirrups should be positioned at 90 degrees in the lithotomy position in order to maximize venous drainage [12]. The authors recommended against abdominal binder use given the potential increased risk of thromboembolism and lack of proven benefit. With regard to compression therapy after body contouring procedures, no published data sets were found on patient outcomes, satisfaction, or complication rate beyond anecdotal reports [35].

*Current Care and Treatment for Thromboembolism Intra- and Post-Operatively *

When thromboembolism occurs, early intervention is required to ensure no further clot progression or embolism to minimize the chance of any associated morbidities. Such morbidities include pulmonary embolisms, post-thrombotic syndrome, right ventricular dysfunction, pulmonary hypertension, recurrence, and perhaps death. This is achieved through numerous types of anticoagulation therapy followed by prophylaxis with clot removal only being considered when the previous options fail to yield beneficial results (Table 3). One of the most common medications used for anticoagulation in the short term is heparins [4]. Unfractionated Heparin (UFH) is one form of anticoagulation that has been found in numerous studies to reduce the chance of DVT, with some stating a reduction from 24.7% in the placebo group to 7.7% in the heparin group with a corresponding reduction in fatal PE from 0.8 to 0.1% [36]. The mechanism behind this reduction is based on the ability of UFH to bind to anti-thrombin III (AT-III). Through this interaction, AT-III has the ability to inhibit certain coagulation factors, especially factor Xa and factor IIa (thrombin). An unfortunate side-effect seen with the use of UFH is Heparin-induced thrombocytopenia (HIT) which is triggered by the immune system’s response to heparin and increases the formation of blood clots [4].

**Table 3:** Pharmacological and non-pharmacological interventions for the treatment of thromboembolism UFH = Ultra-Fractionated Heparin UFH, DVT = Deep Vein Thromboembolism, PE = Pulmonary Embolism, LMWH = Lower Molecular Weight Heparin

Pharmacological			
Study	Year	Treatment	Outcomes
Revilla-Peñaloza et al. [4]	2020	Ultra-Fractionated Heparin	UFH reduce DVT rate incidence from 24.7% in the placebo group to 7.7% in the heparin group, with a corresponding reduction in fatal PE from 0.8 to 0.1%.
Revilla-Peñaloza et al. [4]	2020	Lower Molecular Weight Heparin: Certoparin and Bemiparin	LMWH with a longer half-life compared to UFH prevent DVT in patients with moderate to high thrombogenic risk during the immediate postoperative period with no increase in adverse effects.
Non-Pharmacological			
Klifto et al. [37]	2020	Compression garments	Compressions have decreased the incidence of hemorrhage following breast surgery compared to non-pharmacological prophylaxis.

To combat this potential effect, other heparin-based medications have been developed that have a lesser chance of HIT. One of these alternatives includes certoparin or bemiparin, which is a type of low-molecular-weight heparin (LMWH) [4]. The mechanism of LMWH focuses more on the inhibition of factor Xa however, despite affecting different factors, it has been found to have a similar and, at times, better effect than UFH with a lesser chance of inciting HIT. This was supported by a study that stated that Bemiparin showed greater efficacy when evaluated through venography and a safety profile like UFH [4]. The heparin category of anticoagulants is used for the short term. Other than the heparin group of medications, other medications have been used that target specific coagulation factors, such as fondaparinux, which targets factor Xa, and direct thrombin inhibitors, such as argatroban, which are often switched to after a HIT episode [37]. Klifto et al. also found some benefits with certain non-pharmacological interventions, including mechanism pneumatic compression devices and elastic compression stockings [36].

For long-term anticoagulation and prophylaxis medications such as warfarin have been found to be effective [37]. Warfarin is a type of vitamin K antagonist that blocks the function of vitamin K epoxide reduction in the liver. This depletes the body of the reduced form of vitamin K, which serves as an important cofactor for the gamma-carboxylation of vitamin K depending on coagulation factors such as factors II, VII, IX, and X, as well as coagulation regulatory factors protein C and protein S. Warfarin while used for long term prophylaxis can have early clotting tendencies. The reason for this is due to protein C and protein S have shorter half-lives than the coagulation factors, which can leave one’s body in a hyper-coagulative state. Due to this, warfarin is commonly co-administered with heparin to try to minimize the chance of this type of adverse reaction [37].

Plastic surgeons may be hesitant to administer prophylaxis for venous thromboembolism (VTE) due to the increased risk of hematoma associated with few preventive drugs. Moreover, factors like additional surgical interventions, prolonged hospitalization, increased costs, and the potential flap loss due to hematoma in procedures like free flaps could contribute to this concern. Additionally, the lack of comprehensive studies or information regarding the appropriate dosage of drugs for postoperative prophylaxis highlights the importance of adhering to established guidelines.

## Conclusions

In plastic surgery, deep vein thrombosis and its fatal consequences pose a significant threat to patients. Thus, it is imperative for plastic surgeons to be aware of the risk factors associated with DVT. By incorporating pre-operative risk assessments for all patients, plastic surgeons can assess the risk of thrombosis and decrease the incidence of venous thrombosis by employing appropriate prophylactic measures. It is also crucial for surgeons to be aware of modifications that can be done during surgery, such as positioning the operating room table, and during the postoperative period, such as engaging patients in early ambulation, hydration, and compression devices, all as permitted.
