# Correction: From Risk Assessment to Intervention: A Systematic Review of Thrombosis in Plastic Surgery

**DOI:** 10.7759/cureus.c257

**Published:** 2025-08-26

**Authors:** Heli S Patel, Justin M Camacho, Anastassia Shifchik, Jacob Kalmanovich, Emma Burke, Salam Harb, Alan Patrus, Daniel Cheng, Amir Behnam

**Affiliations:** 1 Allopathic Medicine, Nova Southeastern University Dr. Kiran C. Patel College of Allopathic Medicine, Davie, USA; 2 Department of Medicine, Drexel University College of Medicine, Philadelphia, USA; 3 Medicine, Drexel University College of Medicine, Philadelphia, USA; 4 Allopathic Medicine, Nova Southeastern University Dr. Kiran C Patel College of Allopathic Medicine, Davie, USA; 5 Plastic and Reconstructive Surgery, Tower Health Medical Group, Wyomissing, USA; 6 Plastic and Reconstructive Surgery, Tower Health Medical Group, Wyosmissing, USA

This article has been corrected to fix the following typo and formatting issue in the PRISMA diagram:

"Reports excluded due to irrelevant concept or incorrect thrombosis" has been corrected from 56 to 66 and reformatted for consistency with PRISMA design.

